# Cost-Effectiveness of Electrical Stimulation Therapy in the Treatment of Chronic Wounds: A Systematic Review, Meta-Analysis and Economic Analysis

**DOI:** 10.3390/jmahp13040059

**Published:** 2025-11-24

**Authors:** Jennifer M. Smith, John Posnett, Emma J. Woodmansey

**Affiliations:** 1 JMS Medical Writing Services Ltd., York, UK; 2 Independent Health Economist, York, UK; johnwposnett@gmail.com; 3 Clinical & Scientific Solutions, York, UK; emmajwoodmansey@gmail.com

**Keywords:** chronic wounds, electrical stimulation therapy, cost-effectiveness

## Abstract

Hard-to-heal wounds are a major burden to healthcare systems. Electrical stimulation therapy (EST) is known to improve clinical outcomes, but cost-effectiveness analysis is lacking. The aim was to explore the cost-effectiveness of EST with standard of care (SoC) versus SoC alone. A systematic review and meta-analysis of randomised controlled studies (RCTs) were conducted. Fourteen RCTs were identified, representing 783 patients. EST + SoC, versus SoC alone, significantly increased the proportion of wounds healed (odds ratio [OR] 2.46 [95% CI, 1.75–3.46], *p* < 0.0001) and significantly decreased the mean time to healing (−2.67 weeks (95% CI, 1.49–3.84, *p* < 0.00001). A cost-effectiveness model was developed based on these findings and on the usage and cost of the EST device used in the largest included RCT. Weekly costs of community wound care were taken from published estimates and inflated to 2024 levels, reflecting costs in the UK. In a hypothetical cohort of 100 patients treated over 12 weeks, EST + SoC was estimated to save over GBP 38,000 overall, reduce nursing visits by 385 and lead to 154 more ulcer-free weeks. In conclusion, EST used in the community is a cost-effective addition to SoC with the ability to improve outcomes and reduce human and financial burden of hard-to-heal wounds.

## 1. Introduction

Chronic wounds, for example, venous leg ulcers (VLU), diabetic foot ulcers (DFU) and pressure ulcers (PU) are a major, and growing human, resource and financial burden [1,2] with around 1 million ulcers of the lower limb treated every year in the UK alone, representing around 2% of the adult population [1]. Wounds that failed to heal within 1 year were estimated to cost the UK’s National Health Service (NHS) around GBP 5.6 billion per year, or around 3% of the entire NHS annual budget, based on data from 2017 to 2018 [3]. The financial cost of treating chronic wounds is driven largely by the longer duration of treatment required for a non-healing wounds compared with one that achieves healing [1]. Open wounds need regular dressing changes, pain medication, and regular care visits by healthcare professionals (HCPs), and are at risk of complications such as infection, hospitalisation and amputation, all of which have a cost and resource implication. The longer a wound remains open, the greater the financial and resource burden on healthcare facilities. Advanced techniques, devices or treatments that speed up the time to wound healing and increase the chance of achieving healing may therefore be beneficial, not only to patients but also to healthcare budgets.

Electrical stimulation therapy (EST), including various types and modalities, has been investigated in many randomised controlled trials, and several meta-analyses agree that clinical outcomes are improved with EST compared with standard of care (SoC) alone [4,5,6,7]. Benefits of EST + SoC versus SoC alone, across different types of hard-to-heal wounds, include an increased proportion of wounds which achieve healing, a greater percentage wound area reduction and a faster median time to healing [4,5,6,7]. Within this body of evidence, only a small number of studies have explored the health economic benefits or cost-effectiveness of EST in hard-to-heal wounds and have only done so at a superficial level. Four systematic reviews published by Szoltys-Brzezowska (2023) [8], Borges (2021) [9], Ofstead et al. (2020) [10] and Kawasaki et al. (2011) [11] identified a small number of randomised studies [12,13] or cost-models [14,15,16] which explore the cost effectiveness of EST in chronic wounds [12,13,14,15,16,17]. These systematic reviews all concluded that EST is a cost-effective treatment because the upfront cost of EST devices is more than offset by improvements in healing outcomes, leading to fewer dressing changes and nurse visits, but these observations were based on only a brief description of the supporting evidence. The primary data upon which these statements were based were limited. Three of these studies were published well over a decade ago, meaning the costs of care may no longer be relevant [13,15,16]. In one case, the devices being assessed in a small non-comparative assessment were no longer in commercial use [15]. The final two studies published in 2015 [14] and 2015 [12] were both related to the same portable EST device intended to be used in the community and both presented cost analysis based on costs to the NHS (UK). One retrospectively compared the costs associated with the wound over 12 months before the start of EST with the first 12 months after EST. Results showed that EST yielded a 12% improvement in health gain of 0.09 quality-adjusted life years (QALYs, *p* < 0.01), a 34% reduction in nurse visits and a 26% reduction in the number of dressings. Together this resulted in an 11% reduction in the NHS cost of VLU management over a 12-month period [14]. The second study was an RCT comparing the impact of the same EST device compared with a sham device, in patients with VLU, both applied alongside compression therapy. Results showed that the acquisition cost of the EST increased the costs of care at shorter time horizons (8 weeks), but by 24 weeks following the start of therapy, any additional costs had been offset by a reduction in resource use resulting in lower overall costs of care compared with placebo [12].

Cost-effectiveness in wound care is driven by a combination of the length of time a wound remains open (as open wounds require intervention whereas healed wounds do not) and the proportion of wounds within a cohort who are likely to achieve healing within a particular time frame. However, no meta-analysis was attempted in previous studies to convert such clinical endpoints into cost-effectiveness analysis, and this remains a gap in the current evidence base. Our research hypothesis was that EST would be found to be cost-effective in the treatment of chronic wounds compared to standard care over a time horizon of 12 weeks. The objective of this study was therefore to conduct a systematic review and meta-analysis to explore the proportion of wounds healed and the time to healing for wounds treated with EST + SoC in comparison with SoC only, and to use these findings to explore the cost-effectiveness of EST applied alongside SoC compared with SoC alone.

## 2. Materials and Methods

### 2.1. Systematic Review and Meta-Analysis

A systematic review was conducted according to the PRISMA guidelines [18], to identify randomised controlled trials investigating EST (of any type and parameter) with control treatment. A systematic literature search of PubMed was conducted on 11 April 2025 with the search terms (“electrical stimulation” OR “electric stimulation” OR electroceutical) AND (ulcer OR DFU OR VLU). The use of Pubmed as a single database is in accordance with other systematic reviews in this field [10]. The Pubmed tool was used to limit hits to randomised controlled trials. In addition, previously published meta-analyses of RCTs (see Supplementary Table S1) were explored to identify other studies that may be relevant for inclusion. No limits relating to the date of publication were applied. The review was not registered.

### 2.2. Study Eligibility

Inclusion criteria were as follows: RCTs must have been carried out in human patients with any hard-to-heal wound type (e.g., DFU, arterial, VLU, PU) and treated with any EST modality of any type or parameter, in addition to standard of care (SoC) in one arm and SoC (either SoC only, or SoC with a sham device) in the control arm. A wide definition of SoC was accepted, but treatment must have involved aspects common to present-day SoC, including moist wound healing, debridement, infection control and any gold standard treatments as appropriate (e.g., off-loading for PU and DFU; compression therapy for VLU). Control arm wound management that consisted of wet-to-dry gauze therapy was deemed to not represent present-day SoC, and any papers describing such outdated methods as standard care were excluded. Studies must also have reported at least one of the following endpoints: the proportion of patients healed (in the context of a stated study duration); mean/median time to healing; consumables used; number of study visits required; and any cost-related measures.

Studies were excluded if EST was used for the healing of acute wounds (e.g., burns) or tissues other than skin (e.g., bone repair) or in the absence of a wound (e.g., for pain relief other than wound pain). In vivo/in vitro studies were also excluded. Although meta-analyses, systematic reviews, editorials and general reviews were not included in the systematic review, a search of bibliographies was carried out on highly relevant papers.

Identified articles were manually reviewed for eligibility by one reviewer with appropriate experience and qualifications (qualified to PhD level, with previous experience of conducting systematic reviews). An initial review of titles/abstracts was conducted, and papers which obviously did not satisfy the eligibility criteria were excluded. A further assessment of all remaining papers was conducted, and papers were designated as excluded or included based on the eligibility criteria. Where any uncertainty about the eligibility of a paper existed, the reviewer progressed the paper to the data extraction stage. Papers could be excluded at data extraction stage if they did not contain evaluable and relevant data. Reasons for exclusion were recorded and mirrored the eligibility criteria.

### 2.3. Data Extraction

Data extraction was performed manually by one reviewer into Excel. Where multiple arms were reported in a single study, only the relevant arms were included. In studies where two or more different types of EST, or two or more types of electrode placements were compared with a control arm, data was extracted from the intervention arm that was most clinically relevant. If both intention-to-treat and per protocol data were provided, then intention-to-treat data was used in preference. For studies with multiple timepoints, relevant data from all timepoints was extracted. Other variables, including country of origin, details of EST type/parameters, details of standard wound care, setting, and key patient demographics were also extracted. Missing data was not imputed. No attempts were made to explore additional data from authors of the included papers.

### 2.4. Analysis of Study Quality

Analysis of study quality was conducted by one reviewer using the risk of bias tool embedded in the RevMan software (Cochrane group https://revman.cochrane.org/info, accessed on 12 June 2025). Potential sources of bias included random sequence generation; allocation sequence concealment; blinding of participants/personnel; blinding of outcome assessors; incomplete outcome data; selective outcome reporting. Each of these domains were scored as having either low (green), unclear (amber) or high (red) risk of bias, based on the details presented in each publication.

### 2.5. Statistical Analysis

For continuous data (time to healing) mean differences with 95% confidence intervals (CIs) were expressed. Available data were converted into a consistent unit (weeks) where necessary (days were converted to weeks by dividing by 7; months were converted to weeks by multiplying by 4). If data were not provided in numerical format and only provided in graphs, mean values and standard deviations (SDs) were estimated from the relevant axis of the graphs. In the only case in which this was required, (time to healing endpoint) the week of healing per patient was clearly derived from a Kaplan–Meier chart, allowing calculations of mean (SD) time to healing for each arm of the study. If studies did not provide a mean SD for continuous data, and it could not be derived, but studies did provide medians and interquartile ranges, it was planned to extract medians and estimate the SD as 80% of the interquartile range; however, this conversion was not necessary. The timepoint at which outcome data was collected was expected to vary between studies. Data were broadly grouped by timepoint (less than 8 weeks, 8–16 weeks and greater than 16 weeks). Meta-analysis was considered if there were at least two clinically homogenous studies (defined as studies that investigated the effect of similar interventions on similar participant groups and reported similar outcomes) [19]. In such circumstances the I^2^ statistic was used to quantify the statistical heterogeneity and inform decisions about whether to pool data. I^2^ values ≤ 40% were considered to represent a low level of heterogeneity, >40 to 75% as a moderate level of heterogeneity in the size and direction of the effect and >75%—high level of heterogeneity [19]. Data were not pooled where heterogeneity was very high (I^2^ values of 75% or above). Studies including patients with a variety of chronic wound types (DFU, PU, VLU) and treated with a variety of types of EST, for a variety of treatment durations and follow up periods, were included. All studies with relevant data were considered eligible for inclusion into the meta-analysis. This was deemed to introduce a degree of clinical heterogeneity and therefore data was pooled in all cases using a random effect model. Forest plots were presented, where possible, in the following ways. Dichotomous data (healed/not healed) was assessed via summary estimates as odds ratios (ORs) with 95% CIs. For continuous outcomes (time to healing), the inverse variance method was used when summary estimates were presented as mean differences (MDs) with 95% CIs [19] in line with other systematic reviews in this field [4]. Statistical analysis was conducted using RevMan software (Cochrane group https://revman.cochrane.org/info, accessed on 12 June 2025).

### 2.6. Subgroup Analysis

Subgroup analysis was considered if two or more poolable studies were identified for the following chronic wound types: DFU, PU and VLU [19]. No other sub-group analyses were planned.

### 2.7. Sensitivity Analysis

Sensitivity analysis was conducted [19]. In particular, the impact of the study duration was explored by categorising studies into the following groups: those with <8-week duration; 8–16-week duration; >16–24-week duration and those with >24-week duration or longer. This was necessary as the proportion of patients achieving healing would be expected to increase as the study duration increased, and so accounting for study duration may have reduced statistical heterogeneity.

### 2.8. Cost Effectiveness Analysis

Evidence obtained from the meta-analysis was used to inform an assessment of the cost-effectiveness of EST + SoC compared with SoC alone. Cost-effectiveness was assessed by comparing expected costs and outcomes for a hypothetical cohort of 100 patients with a hard-to-heal wound treated with EST + SoC or with SoC alone. The time horizon of the analysis was 12 weeks, consistent with the literature identified in the systematic review. Outcomes were measured by the number of wound-free weeks in a 12-week period, estimated from the proportion of ulcers healed and the average time to healing. Values of these variables were derived from the results of the meta-analysis. Costs were measured weekly and reflect the average cost to the UK National Health Service (NHS) of treating a patient with a chronic wound in a mixed acute/community setting. The average weekly cost of GBP 403.69 includes nurse time, GP and outpatient appointments, dressings and other materials. The cost is a weighted average of treatment costs for hard-to-heal wounds derived from a previous estimate of the annual NHS cost of managing a range of different wound types [20]. Annual costs were adjusted to weekly costs assuming an average treatment period for a chronic wound of 14 weeks, which was the average treatment period (3.5 months) in the control group of a large RCT comparing EST with control in patients with a VLU [12]. The original estimates of cost at 2013/14 prices [20] were updated to reflect 2023/24 values by applying the NHS Cost and Inflation Index [21]. Estimates of nurse time assume an average of 2.5 patient visits per week (5 per 14 days) at 18 min per visit [22]. The cost of the modelled EST device was based on the June 2025 Drug Tariff cost (GBP 240) of Accel-Heal Solo (Accel-Heal Technologies Ltd., Kent, UK) assuming one application per patient, consistent with the recommended use of the device. The UK Drug Tariff (Part IXA-Appliances) lists just two EST devices for wound healing and both have a similar price. Results are therefore generalisable for the current UK NHS, but may differ for devices with different prices and/or characteristics. This device is designed to be used alongside standard wound dressings in the patient’s home and does not require additional nurse visits. The main drivers of costs and outcomes are the proportion of ulcers healed and the time to healing. To test the robustness of results, assumptions about time to healing and the proportion healed were varied in a two-way sensitivity analysis.

## 3. Results

### 3.1. Systematic Review

**Figure 1:**
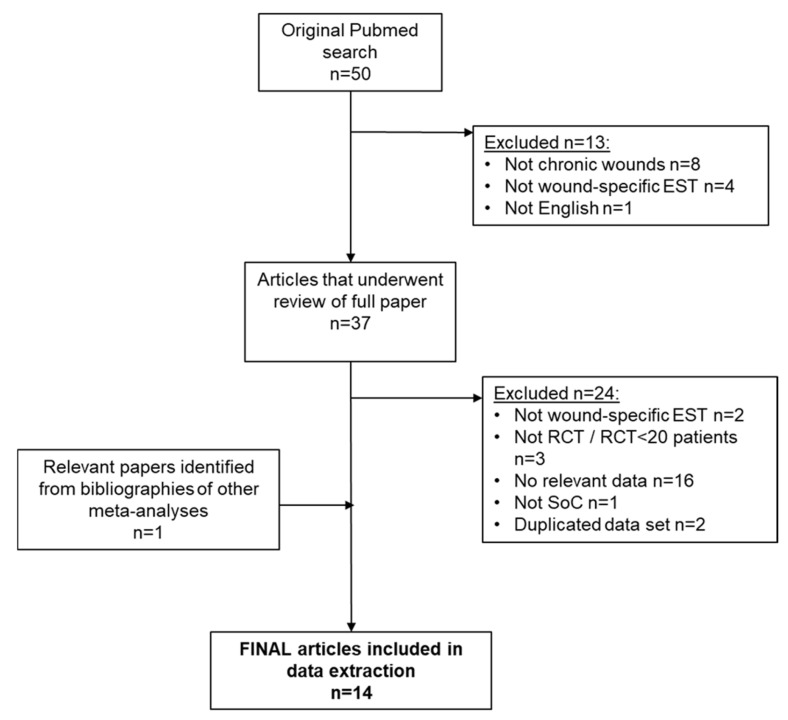
Systematic review flow chart.

The literature search identified 50 studies. Of these, 13 satisfied the inclusion and exclusion criteria and were included in the review. One additional study was identified from other sources and was also included, making a total of 14 papers eligible for the meta-analysis [12,13,23,24,25,26,27,28,29,30,31,32,33,34], representing 783 patients. A flow chart showing study inclusion and exclusion is shown in Figure 1. The 14 included studies are described in Table 1.

**Figure 2:**
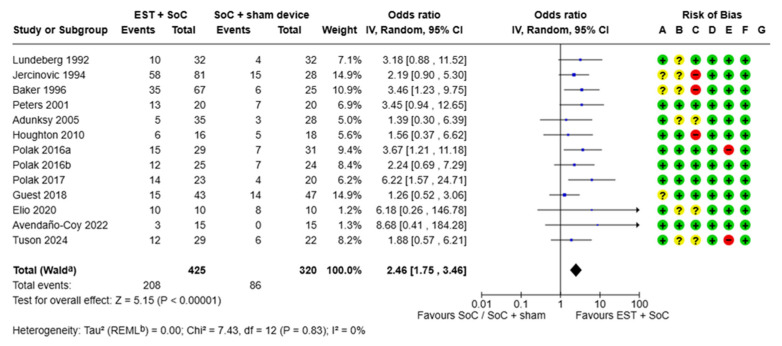
Forest plot showing proportion of wounds healed. ^a^ CI was calculated by the Wald-type method; ^b^ Tau^2^ was calculated by the Restricted Maximum-Likelihood method. Risk of bias included random sequence generation (selection bias, A), allocation concealment (selection bias, B), blinding of participants and personnel (performance bias, C), blinding of outcome assessment (detection bias, D), incomplete outcome data (attrition bias, E), selective reporting (reporting bias, F) and other bias (G). CI—confidence interval; EST—electrical stimulation therapy; IV—inverse variance; SoC—standard of care [12,23,24,25,26,27,28,29,30,31,32,33,34].

**Figure 3:**
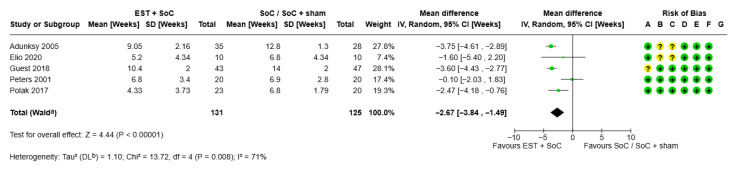
**Forest plot showing time to healing for chronic wounds.** ^a^ CI was calculated by the Wald-type method; ^b^ Tau^2^ was calculated by the Restricted Maximum-Likelihood method. Risk of bias included random sequence generation (selection bias, A), allocation concealment (selection bias, B), blinding of participants and personnel (performance bias, C), blinding of outcome assessment (detection bias, D), incomplete outcome data (attrition bias, E), selective reporting (reporting bias, F) and other bias (G). CI—confidence interval; EST—electrical stimulation therapy; IV—inverse variance; SD—standard deviation; SoC—standard of care [12,25,26,30,31].

Many potential aspects of study bias were addressed through the methodologies employed; however, some concerns remained related to the lack of blinding to allocation (performance bias) and lack of measures taken to reduce allocation concealment (selection bias). For studies using microcurrent EST devices (delivering stimulation below patients’ sensory threshold) in comparison with sham devices [12,24], performance bias was of minimal concern, whereas with devices compared with no sham device, or where the stimulation could be perceived by the patients (as with high voltage pulsed current [HVPC] and neuromuscular electrical stimulation [NMES] type EST parameters), blinding of the patients and caregivers was not possible. Attrition bias was an issue with two studies due to reporting of the per protocol population as opposed to the intent-to treat population [23,27]. Because the outcomes of interest here were based on a fundamental, objective and dichotomous clinical outcome (healed/not healed), we found minimal bias relating to detection bias or selective reporting. For details of study bias, refer to Figure 2 and Figure 3.

#### 3.1.1. Proportion of Chronic Wounds Healed

In total, 13 studies reported the proportion of wounds healed. Eight studies reported outcomes in patients with PU [24,26,27,28,29,30,32,33], three studies in VLU [12,23,25] and two studies in DFU [31,34]. Statistical heterogeneity in terms of the magnitude of the difference between control and EST arms was negligible (I^2^ = 0). Across all studies, wounds treated with EST + SoC were almost 2.5 times more likely to achieve healing compared with wounds treated with SoC (either alone or with sham device; OR 2.46 [95% CI, 1.75–3.46], *p* < 0.0001, Figure 2). The percentage of wounds overall which achieved healing within the study duration (which ranged from 3.5 weeks to 52 weeks but with a median of 12 weeks) was 48.9% for those treated with EST + SoC versus 26.9% for those treated with SoC with a sham device.

The study duration for these studies ranged from 3.5 weeks to 52 weeks. This wide range of study durations may have introduced significant variability and so a sensitivity analysis was conducted to account for studies with very short (<8 weeks) [4,27,28,32] and very long (>20 weeks) [24] durations. The eight remaining studies had study durations of 8–16 weeks. The sensitivity analysis showed that removing the studies with a very short or very long study duration did not meaningfully affect the findings compared with the overall findings shown in Figure 2 (Supplementary Table S2). In studies with durations of between 8 and 16 weeks, patients treated with EST + SoC were 2.3 times more likely to heal than wounds treated with SoC (either alone or with sham device; OR 2.30 [95% CI 1.44–3.69], *p* = 0.0005). This analysis suggested that inclusion of all studies, regardless of study duration was appropriate. Another potential source of heterogeneity was the aetiology of hard-to-heal wounds included in each study. A sub-analysis looking separately at different wound types identified similar findings in PU (2.75 [95%CI 1.78, 4.25], *p* < 0.00001), VLU (1.56 [95%CI 0.78–3.12], *p* = 0.21) and DFU (3.31 [95%CI 1.33–8.26] *p* = 0.01) (Supplementary Table S3), suggesting that inclusion of all studies, regardless of aetiology, was appropriate.

#### 3.1.2. Time to Healing for Chronic Wounds

Five RCTs reported time to healing [12,25,26,30,31]. Results are shown in Figure 3. Briefly, 4/5 studies showed that EST + SoC accelerated the time to healing and one study (40-patient study in DFU) showed no significant difference. Overall, mean time to healing was calculated as 10.93 weeks for SoC alone versus 8.26 weeks for EST + SoC, representing a 24% shorter time to healing. Mean overall healing occurred 2.67 (95% CI, 1.49 to 3.84) weeks faster with EST + SoC than with SoC (with or without sham device). The difference was statistically significant (*p* < 0.00001). These studies represented VLU [12,25], PU [26,30] and DFU [31]. All studies assessed a time horizon of between 8 and 16 weeks (median study duration was 12 weeks) which was considered an appropriate time range for pooling of results.

Sensitivity analysis showed that one study conducted on DFU (Peters et al., 2001) [31] was responsible for the statistical heterogeneity (removing this study decreased the I^2^ value from 71% to 0% and the difference in time to healing increased to 3.5 weeks [95% CI 4.06–2.94 weeks] faster with EST + SoC vs. SoC alone); however, since there was no legitimate reason to exclude this study, it was retained in the analysis. Although this study did not show a difference in time to healing for DFU, it did show that more patients treated with EST + SoC healed compared with those treated with SoC alone (65% vs. 35%, respectively, Figure 2) thus representing a benefit for EST in DFU. Because of the relatively low numbers of studies reporting this outcome, no other sensitivity analyses were conducted. The high I^2^ value in this analysis reduces the robustness of the finding.

Although Tuson et al. (2024) [23] reported the mean time to healing, no variability was provided and so this could not be added to the meta-analysis and is not shown in the forest plot. Data from this paper did confirm the general findings of the meta-analysis, that treatment with EST did result in faster healing compared with SoC only (mean time to healing of 25.3 vs. 37.6 weeks, respectively).

### 3.2. Cost Effectiveness of EST

Table 2 summarises the results of the cost-effectiveness modelling. Expected costs and outcomes were estimated using values from the meta-analysis for all chronic wounds. The proportion of wounds healed (Figure 2) with EST plus SoC vs. Soc was 48.9% vs. 26.9% (mean difference 22%). Mean time to healing (Figure 3) for EST plus SoC versus SoC was 8.26 weeks versus 10.93 weeks (mean difference, 2.67 weeks). The addition of EST to SoC in patients with chronic wounds is expected to improve patient outcomes. In the economic model, shorter time to healing and a greater proportion of ulcers healed within the 12-week period led to an extra 154 ulcer-free weeks per 100 patients treated, equivalent to 1.54 weeks per patient. Total costs are lower with EST by GBP 38,226 (GBP 382 per patient) because the number of weeks of treatment required is lower with the addition of EST. Faster healing times reduce the number of nurse visits by 385 per 12-week period for every 100 patients treated, a potential saving in nurse time of 116 h. The addition of EST to SoC produces better patient outcomes (more weeks free of ulcer) and lower costs. On this basis, EST is a cost-effective addition to SoC.

A sensitivity analysis varied assumptions about time to healing and the proportion of ulcers healed with the addition of EST (Table 3). The base-case analysis assumed a mean improvement in time to healing with the addition of EST of 2.67 weeks compared with SoC alone. The 95% confidence interval around this central estimate (−1.49 to −3.94) reflects the uncertainty around the estimate. The sensitivity analysis re-estimated costs and outcomes assuming the reduction in time to healing with EST was 1.49 weeks or 3.94 weeks rather than 2.67 weeks. The sensitivity analysis also varied the difference in the proportion healed at 12 weeks with the addition of EST. The base-case assumed a difference of 22% (48.9–26.9%). The sensitivity analysis re-estimated costs and outcomes assuming the magnitude of the difference between study arms was reduced by half (to 11%), three-quarters (to 5.5%) or where there was no difference in the proportion of patients healed (0%). Table 3 shows the effect on expected costs. Costs remained lower with the addition of EST to SoC in all of the modelled cases and EST + SoC remained a cost-effective alternative to SoC. Lower costs were driven by a shorter duration of treatment, and this was also associated with patient benefits in the form of more ulcer-free weeks.

## 4. Discussion

Analysis based on this systematic review and meta-analysis suggests that the addition of EST to SoC is clinically effective due to its ability to significantly increase the proportion of hard-to-heal wounds which achieve healing, along with its ability to accelerate the time to healing compared to SoC alone. This combination of benefits has been shown to lead to a reduction in the caseload of patients with open wounds who need regular visits [35], dressing changes [36] and pain relief [36,37], all of which have cost implications, and an increase in patients with healed wounds [6,7,38] which need fewer/no regular visits and limited/no wound related consumables. The cost-effectiveness analysis showed that at or below a particular price point, EST used in the community is expected to be cost-effective over a 12-week time horizon.

The use of EST in non-healing wounds is supported by an extensive body of clinical evidence. This evidence base consisting of over 40 RCTs, with many supporting meta-analyses (Supplementary Table S1) and systematic reviews contains relatively little cost-effectiveness analysis. Two RCTs comparing EST with SoC directly reported health economic outcomes when used in chronic wounds [12,13]. One study had limited applicability—EST was found to increase the cost of wound management but the authors noted the particularly high price of the device being tested, which is not representative of the typical price point of EST; data from this study was also collected in the 1990s, and the cost-related aspects may have minimal relevance now [13]. The second study was reported in 2018, conducted in VLU using a modern type of EST and was considered highly relevant [12]. Clinical data from both of these studies were included in the meta-analysis presented here. In a third RCT in acute abdominal wounds, EST was found to lower the costs of wound management slightly compared with NPWT in a hospital setting but was this study was not directly applicable to community-based care of hard-to-heal wounds and did not compare EST to SoC, so it was not deemed relevant for inclusion in this meta-analysis [17].

The results of the meta-analysis found that the addition of EST to SoC could significantly increase the proportion of patients who achieve healing by 2.5 times and reduce time to healing by an average of 2.67 weeks, compared with SoC alone. Despite the clinical heterogeneity described in the included studies (different wound types, durations of treatment, follow up, and types of EST) the statistical heterogeneity (I^2^) was generally low, meaning that the findings are generalizable across all of these variables. Over a 12-week period, patients treated with EST at or below the modelled cost would be expected to have an average of 1.54 additional weeks ulcer-free, with costs to the healthcare system expected to be lower by GBP 382 per patient. For every 100 patients treated over a 12-week time horizon, estimated cost savings amount to more than GBP 38,000. Improved healing times also reduced the burden on nurse time by reducing the number of weeks of treatment. Over a 12-week period, the addition of EST to SoC was estimated to release almost 120 h of nurse time for other patient-centred activities, compared to SoC alone. The sensitivity analysis explored a range of scenarios and found that even when there was no difference in the proportion of wounds healed, as long as there was a benefit in the time to healing, EST treatment would be expected to be cost-effective (Table 3).

Results of the cost-effectiveness analysis are naturally subject to uncertainty because patient outcomes and costs vary between healthcare organisations depending on local factors. Patients treated in the real-world may also differ from populations eligible for enrolment into randomised clinical trials. Varying the values of key variables within a plausible range in a sensitivity analysis is a way to assess the robustness of the results of the analysis. Varying both the proportion of ulcers healed and time to healing, the addition of EST remained cost-effective in all of the scenarios assessed.

This study had several limitations. Firstly, some relevant data could not be included into the meta-analysis because of the lack of specific information presented in the source paper, for example, lack of variability around data points, lack of clear expression of the duration of study, or unclear assignment of patients to different arms of the study. No assumptions or imputations were made where data were missing. This slightly reduced the number of papers eligible for inclusion. Secondly, there were several major sources of heterogeneity between studies, including wound types, and the study duration of the different studies. These differences were explored in sensitivity analyses, but these analyses were limited because the numbers of papers for each specific scenario were low, specifically DFU and VLU. In particular, more randomised studies exploring patients with DFU and VLU are needed to strengthen the evidence base. However, the low statistical heterogeneity observed in the analysis suggested that the effect of EST was consistent across different wound types and different types of EST, reducing concern about these potential sources of heterogeneity. Other potential sources of heterogeneity, such as patient characteristics, or type or parameters of EST, were not explored and this may be a valuable approach for future studies. Many of the eligible studies were based on relatively small enrolled populations, with uncertain power, meaning that the meta-analysis may be underpowered. The time horizon of the cost-effectiveness analysis was limited to 12 weeks as this was the most appropriate time horizon given the identified studies. Chronic wounds often persist for more time or might even recur. Longer-term modelling, including analysis of wound recurrence may affect conclusions; further research would be required to consider these points. The cost-effectiveness analysis was limited to the perspective of the NHS (UK). Other healthcare systems in other countries may have different outcomes. Although the meta-analysis was device agnostic (including many different types of devices with a variety of delivery methods), the cost-effectiveness model is valid for a device used in a community setting and a cost of GBP 240 per patient or less. GBP 240 is the average cost of both the EST devices listed on the UK Drug Tariff. Devices with higher price points, different comparators used in routine clinical practice, or different delivery models (e.g., a requirement for additional in-person clinic appointments) will affect estimates of cost-effectiveness.

## 5. Conclusions

This meta-analysis of randomised controlled trials comparing EST + SoC with SoC alone confirmed a significant 2.5-time increase in the proportion of patients achieving healing over an 8–16-week time horizon, as well as the mean time to healing being on average 2.7 weeks faster than with SoC alone. These clinical findings across different types of hard-to-heal wounds, including VLU, DFU and PU, suggest that patients would benefit from the addition of EST to existing SoC. A cost-effectiveness analysis based on the findings of the meta-analysis showed that the addition of EST to standard care is expected to improve outcomes for patients and reduce costs for the NHS.

## Figures and Tables

**Table 1:** Details of relevant papers identified by the systematic review.

Paper	Wound Type	N Patients	Study Duration	Experimental/Control Arm	Proportion of Wounds Healed, %	Mean (SD) Time to Healing, Weeks	Cost Analysis
Tuson et al. (2024) [23]	VLU	51	16 weeks	NMES + SoC	42%	25.3	N
				SoC only	27%	37.6	
Avendano-Coy (2022) [24]	PU (nursing care setting)	30	25 days (3.5 weeks)	Microcurrent EST + SoC	20%	NR	N
				Sham device + SoC	0%	NR	
Elio (2020) [25]	VLU	30	8 weeks	REAC + SoC	100%	5.2 (4.3)	N
				SoC only	80%	6.8 (4.3)	
Guest (2018) [12]	VLU	90	8, 16 and 24 weeks	Microcurrent EST + SoC	34%	10.4 (2.0)	Y
				Sham device + SoC	30%	14.0 (2.0)	
Polak (2017) [26]	PU (nursing care)	63	12 weeks	HVPC + SoC	60.9%	4.3 (3.7)	N
				Sham device + SoC	20.0%	6.8 (1.8)	
Polak (2016a) [27]	PU (nursing care)	77	6 weeks	HVPC + SoC	51.7%	NR	N
				Sham device + SoC	22.6	NR	
Polak (2016b) [28]	PU (nursing care)	49	6 weeks	HVPC + SoC	48.0%	NR	N
				Sham device + SoC	29.2%	NR	
Houghton (2010) [29]	PU (spinal cord injury)	34	12 weeks	HVPC + SoC	37.5%	NR	N
				Sham device + SoC	27.8%	NR	
Junger (2008) [13]	VLU	39	20 weeks	300 μA EST + SoC	NR	NR	Y
				Sham device + SoC	NR	NR	
Adunsky (2005) [30]	PU	63	8–12 weeks	DC EST	14.0%	9.1 (2.2)	N
				Sham device	10.7%	12.8 (1.3)	
Peters (2001) [31]	DFU	40	12 weeks	PC (Micro-Z) + SoC	65.0%	6.8 (3.4)	N
				Sham device + SoC	35.0%	6.9 (2.8)	
Baker (1996) [32]	PU (spinal cord injury)	80 *	4 weeks	EST (biphasic pulsed current)	52.2%	NR	N
				Sham device	24.0%	NR	
Jercinovich (1994) [33]	PU (spinal cord injury)	73 **	52 weeks	EST (NMES type) + SoC	71.6%	NR	N
				SoC	53.6%	NR	
Lundeberg (1992) [34]	DFU	64	12 weeks	EST (AC)	31.3%	NR	N
				Sham device	12.5%	NR	

All studies were randomised controlled trials with 20 or more subjects. * 80 patients had 92 PU treated for 4 weeks. ** 73 patients had 109 pressure ulcers, treated for 4 weeks and followed for 52 weeks. In both * and ** the proportion of wounds healed was expressed as % of PU, not % of patients. AC—alternating current; DC—direct current; DFU—diabetic foot ulcer; EST—electrical stimulation therapy; HVPC—high-voltage pulsed current; NMES—neuromuscular electrical stimulation; PU—pressure ulcer; SoC—standard of care; VLU—venous leg ulcer; Y—yes.

**Table 2:** Cost-effectiveness of adding EST to SoC for the treatment of chronic wounds.

		SoC	EST + SoC	EST Incremental
Cohort size, patients treated		100	100	
Time period modelled, weeks		12	12	
Patients healed, % *		26.9	48.9	
Time to heal, weeks *		10.93	8.26	
Cost of SoC, GBP per week ^		403.69	403.69	
Cost of EST †		0	GBP 240.00	
Number of nursing visits per week		2.5	2.5	
Time/visit, min		18	18	
Resource and cost differences between arms based on treating 100 patients over a 12-week period				
Weeks of treatment	Wounds that healed	293.74	403.91	
	Wounds not healed	877.50	613.20	
	Total	1171.24	1017.11	−154.13
Weeks free of ulcer	Total	28.76	182.89	154.13
Costs, GBP	Cost of SoC	GBP 472,825	GBP 410,599	
	Cost of EST	0	GBP 24,000	
	Total	GBP 472,825	GBP 434,599	−GBP 38,226
Resources	Treatment weeks	1171.24	1017.11	
	Number of visits	2928	2543	−385
	Time required for visits, hours	878	763	−116

* The proportion of wounds healed and the time to healing were derived from the meta-analysis (Figure 2 and Figure 3); ^ costs of care were based on a hypothetical cohort of 100 patients followed over a 12-week time horizon; weekly costs of treatment were derived from the literature as described in the Methods section and adjusted to reflect 2023/24. † Costs of EST reflect 12 days of treatment with a single-use automated microcurrent EST device in line with its instructions for use. EST—electrical stimulation therapy; SoC—standard of care.

**Table 3:** Sensitivity analysis varying the difference between SoC and EST + SoC in time to healing and healing proportion.

Reduced Time to Healing as a Result of Adding EST to SoC *	Additional % Healed with Addition of EST to SoC ^			
	22%	11%	5.5%	0%
2.67 weeks faster	GBP 38,226 savings	GBP 33,464 savings	GBP 31,088 savings	GBP 28,712 savings
1.49 weeks faster	GBP 25,424 savings	GBP 15,409 savings	GBP 10,414 savings	GBP 5418 savings
3.94 weeks faster	GBP 50,919 savings	GBP 51,365 savings	GBP 51,587 savings	GBP 51,809 savings

* Time to healing was taken from the meta-analysis (Figure 3), representing the mean estimate (2.67 weeks faster: the base case), the lower 95% CI of the estimate (1.49 weeks faster) and the upper 95% CI (3.94 weeks faster). ^ The additional proportion of wounds healed was derived from the meta-analysis (Figure 2, 22%: the base case). This assumption was varied to the detriment of EST, by assuming that the magnitude of the effect was half or three-quarters the magnitude of the base case (11%, 5.5%, respectively) or that there was no increase in proportion of patients healed with EST + SoC vs. SoC alone (0%). Positive values represent cost savings over a 12-week time horizon as a result of adding EST to SoC.

## Data Availability

The original contributions presented in this study are included in the article/Supplementary Materials. Further inquiries can be directed to the corresponding author.

## Supplementary Materials

The following supporting information can be downloaded at https://www.mdpi.com/article/10.3390/jmahp13040059/s1, Table S1: The meta-analyses of EST published to date. Table S2: Results of a sensitivity analysis reducing the heterogeneity of study durations eligible for inclusion into the analysis. Table S3: Results of a sensitivity analysis to explore the impact of inclusion of different wound types into the analysis (divided into pressure ulcer, venous leg ulcer or diabetic foot ulcer) [4,5,6,7,38,39,40,41,42,43,44,45,46].

## Abbreviations

The following abbreviations are used in this manuscript:

CI	Confidence interval
DFU	Diabetic foot ulcer
EST	Electrical Stimulation Therapy
GP	General practitioner
HCP	Healthcare professional
NHS	National Health Service
OR	Odds ratio
PU	Pressure ulcer
RCT	Randomised controlled trial
SoC	Standard of care
VLU	Venous leg ulcer
