# Supplementary material for: Cost-Effectiveness of Electrical Stimulation Therapy in the Treatment of Chronic Wounds: A Systematic Review, Meta-Analysis and Economic Analysis
Source: J Mark Access Health Policy. 2025 Nov 24;13(4):59. doi: 10.3390/jmahp13040059 (PMC12734067; doi:10.3390/jmahp13040059)
Supplement: Supplementary file 1 [file jmahp-13-00059-s001.zip › jmahp-3869486-supplementary.pdf]

Supplemental Table S1: . The meta-analyses of EST published to date.

Citation	Year	Wound type	Top level findings
Lan et al. [6]	2024	DFU	Increased rate of healing and greater proportion of healed ulcers with EST vs control/placebo
Zheng et al. [38]	2022	DFU	Significantly greater ulcer reduction; healing rate
Chen et al. [39]	2023	PU	EST is a relatively effective and safe adjunctive therapy for PU treatment.
Girgis et al. [40]	2023	DFU and PU	Although conclusive evidence regarding the effect of HVMPC on DFU was borderline (more evidence needed) collateral evidence might suggest a potential benefit. Direct evidence, with moderate certainty, may support its efficacy in treating PrUs.
Avendaño-Coy et al. [4]	2021	Any chronic ulcers	Focussed on microcurrent EST. 8 RCTs analysed. EST decreases wound area and decreased pain vs standard care
Arora et al. [7]	2020	Pressure ulcer	Cochrane review. ES probably increases the proportion of pressure ulcers healed and the rate of pressure ulcer healing (moderate certainty evidence),
Chen et al. [5]	2020	DFU	7 RCTs analysed. Faster healing at 4- and 12-weeks with EST vs control.
Girgis et al. [41]	2018	Pressure ulcer	EST decreases wound area; EST increases chance of healing
Khouri et al. [42]	2017	Various chronic wounds	This study confirms the overall efficacy of ES to enhance healing of chronic wounds
Lala et al. [43]	2016	Pressure ulcer (spinal cord patients)	EST decreases wound area; EST increases chance of healing
Liu et al. [44]	2016	Pressure ulcer	EST increases rate of healing

Citation	Year	Wound type	Top level findings
		(spinal cord patients)	
Barnes et al. [45]	2014	Chronic ulcers	EST decreases wound area
Gardner et al. [46]	1999	Chronic ulcers	EST increases rate of healing

Supplemental Table S2. Results of a sensitivity analysis reducing the heterogeneity of study durations eligible for inclusion into the analysis. The sensitivity analysis confirms the expectation that addition of EST to SoC will improves the proportion of patients who achieve healing.

Criteria	N studies (n patients)	OR (95% CI)	*p*-value
Overall	13 (745)	2.46 (1.75-3.46)	p<0.00001
Removed high and low duration studies*	8 (405)	2.30 [1.44 , 3.69]	p=0.0005

Supplemental Table S2. Sensitivity analysis. **studies with duration shorter than 8 weeks or longer than 24 weeks were excluded; all remaining studies had durations of 8-16 weeks. The overall finding remained similar meaning that use of all time horizons into the main analysis was appropriate.*

Supplemental Table S3. Results of a sensitivity analysis to explore the impact of inclusion of different wound types into the analysis (divided into pressure ulcer, venous leg ulcer or diabetic foot ulcer). The sensitivity analysis confirms the expectation that addition of EST to SoC will improves the proportion of patients who achieve healing, regardless of specific aetiology.

Criteria	N studies (n patients)	OR (95% CI)	*p*-value
Overall	13 (745)	2.46 (1.75-3.46)	p<0.00001
PU	8 (480)	2.75 [1.78 , 4.25]	p<0.00001
VLU	3 (161)	1.56 (0.78-3.12)	p=0.21
DFU	2 (104)	3.31 (1.33-8.26)	p=0.01

Supplemental Table S3. Sensitivity analysis. **studies recruiting different hard-to-heal wound types (PU, DFU, VLU) were explored separately. The overall finding remained similar meaning that aggregation of all wound types into the main analysis was appropriate.*
